# Efficacy and Safety of *Lactobacillus delbrueckii* subsp. *lactis* CKDB001 Supplementation on Cognitive Function in Mild Cognitive Impairment: A Randomized, Double-Blind, Placebo-Controlled Clinical Trial

**DOI:** 10.3390/nu17203313

**Published:** 2025-10-21

**Authors:** Hyang-Im Baek, So-Young Kwon, Hye-Ji Noh, Su Young Son, Jong Cheon Joo, Soo Jung Park

**Affiliations:** 1 Department of Food Science & Nutrition, Woosuk University, Wanju 55338, Republic of Korea; hyangim100@gmail.com; 2 Research Institute, Chong Kun Dang Bio (CKD Bio), Seoul 03742, Republic of Korea; ksy2022@ckdbio.com (S.-Y.K.); nhj@ckdbio.com (H.-J.N.); 3 Genome & Company, Suwon 16229, Republic of Korea; suyoung@genomecom.co.kr; 4 Department of Sasang Constitutional Medicine, College of Korean Medicine, Wonkwang University, Iksan 54538, Republic of Korea; 5 Department of Sasang Constitutional Medicine, College of Korean Medicine, Woosuk University, Jeonju 55338, Republic of Korea

**Keywords:** mild cognitive impairment, cognitive function, probiotics, *Lactobacillus delbrueckii* subsp. *lactis* CKDB001, functional food, clinical trial

## Abstract

Background: Modulation of the gut–brain axis using probiotics present a promising approach for enhancing cognitive function in mild cognitive impairment (MCI). In prior non-clinical research, *Lactobacillus delbrueckii* subsp. *lactis* CKDB001 (LL) exhibited potential to enhance cognitive function. We therefore conducted a clinical trial to assess the efficacy and safety of LL supplementation in MCI. Methods: A 12-week, randomized, double-blind, placebo-controlled, multi-center trial was performed in 100 participants aged 55–80 years. Subjects were randomly assigned to receive LL (*n* = 50, 5.0 × 10^9^ CFU/day) or placebo (*n* = 50). Efficacy and safety were evaluated at baseline and after 12 weeks. Results: LL supplementation resulted in significantly greater improvements than placebo in the Alzheimer’s Disease Assessment Scale–Cognition 13 total score, the memory sub-score, reaction time for Part A of the Trail Making Test, and word/color reaction times on the Stroop test. Taxonomic and metabolomic profiling of fecal samples showed significantly greater changes in the relative abundance of beneficial microorganisms in the LL group, with the most pronounced shifts at the family (*Lactobacillaceae*, *Bifidobacteriaceae*) and genus (*Lactobacillus*) levels. In addition, the LL group exhibited significantly higher fecal levels of indole-derived metabolites, including 5-hydroxyindole-3-acetic acid, indole-3-lactic acid, and indole-3-glycol. Safety assessments indicated LL was safe and well-tolerated, with no clinically relevant changes in laboratory findings or adverse events. Conclusions: These findings suggest that LL supplementation may enhance cognitive function in MCI by modulating the gut–brain axis through effects on gut microbiota and related metabolites, and could serve as a safe functional food to support cognitive health.

## 1. Introduction

The global population of older adults is rising at a rapid pace, with the number of individuals aged 60 years and older reaching 1 billion in 2021 and projected to reach 2.1 billion by 2050 [1]. Aging may negatively impact the nervous system, which can result in cognitive decline and, in severe cases, dementia [2]. Concurrently, the prevalence of dementia has increased markedly [3]. In 2021, it was estimated that 57 million people worldwide were affected by dementia, and over 10 million new cases were diagnosed annually [4]. Dementia is one of the leading causes of death and disability among older adults, and its global public health and economic burden has increased substantially over the past few decades, with projections indicating further increases as the population ages [4,5]. This underscores the urgent need to develop effective prevention and management strategies to support healthy aging and alleviate the extensive societal and financial consequences of dementia [3,6].

Alzheimer’s disease (AD) is the most prevalent neurodegenerative disorder, accounting for about 60–80% of all dementia cases [7]. The disease is marked by progressive deterioration of cognitive and functional capacity, neuropsychiatric symptoms, increased caregiver burden, and premature mortality [8]. Once the disease develops, delaying its progression is highly challenging. Although approved amyloid-targeting antibody therapies may modify the disease course, the majority of available AD drugs still provide symptomatic relief rather than a fundamental cure. Therefore, preventing the onset of AD is considered the most effective approach to reducing its overall burden [9].

Mild cognitive impairment (MCI) is a pre-dementia stage characterized by cognitive decline greater than expected for normal aging, yet not severe enough to significantly interfere with daily functioning [10]. Previous studies have shown that individuals with MCI have an increased risk of developing dementia, especially AD [11,12]. Each year, approximately 1–2% of cognitively healthy older adults progress to AD, whereas 10–15% of individuals with MCI convert annually [13]. Because MCI represents a high-risk state for AD, interventions aimed at improving cognitive function in this population may help prevent or delay disease onset [14]. Notably, some individuals with MCI revert to normal cognitive status [15,16]. Consequently, the timely implementation of suitable interventions during the MCI stage is crucial for mitigating the future burden of dementia [9].

Recently, numerous studies have highlighted the correlation between the human gut microbiota and neurodegenerative diseases [17,18,19,20]. The gut and brain communicate through the gut–brain axis, which is a bidirectional pathway through which bacterial metabolites can exert either beneficial or harmful effects on the central nervous system [21]. An imbalance in the gut microbial community, termed gut dysbiosis, together with alterations in the gut–brain axis, has been linked to the initiation and progression of AD [22]. Probiotics are live microorganisms that, when provided in sufficient quantities, confer health benefits and have received considerable interest for their capacity to support a balanced gut microbiota [23]. Studies indicate that probiotics may aid in reestablishing microbial equilibrium, mitigating AD progression of AD, and ameliorating cognitive impairment, thus providing beneficial effects that extend beyond gastrointestinal well-being [24]. With continued progress in research on the gut–brain axis, probiotics that modulate gut microbiota composition are increasingly recognized as a potential approach for preventing and managing AD and dementia [6].

*Lactobacillus delbrueckii* subsp. *lactis* CKDB001 (LL) is a probiotic strain isolated from fermented milk, which has demonstrated beneficial effects on hepatic lipid metabolism and enhancement of fatty acid oxidation in non-clinical studies [25,26]. Additionally, it has been reported to modulate gut microbiota composition by increasing beneficial bacteria, reducing pro-inflammatory genera, suppressing lipopolysaccharide biosynthesis, and enhancing carbohydrate metabolism and short-chain fatty acid production [27]. Moreover, recent non-clinical studies suggest that microbiome-derived indole-3-lactic acid (ILA), produced by LL, can activate the aryl hydrocarbon receptor (AhR), mitigate amyloid pathology, and thus improve cognitive function, providing a potential mechanistic link between modulation of the gut microbiota and neuroprotection [28].

Although these cognitive and gut microbiota-modulating effects have been demonstrated in experimental models, evidence in humans is still scarce. Accordingly, this randomized, double-blind, placebo-controlled, multicenter trial aimed to evaluate the efficacy and safety of LL for cognitive function in older adults with MCI, while exploring gut microbiota and related metabolite changes.

## 2. Materials and Methods

### 2.1. Ethics Statement

The study protocol (Protocol No: CKDB001-02) and informed consent form were reviewed and approved by the Institutional Review Board (IRB) of Wonkwang University Korean Medicine Hospital, Jeonju (IRB approval No.: WUJKMH-IRB-2023-0011; approval date: 20 November 2023), and Woosuk University Korean Medicine Hospital (IRB approval No.: WSOH IRB H2311-04; approval date: 14 December 2023). The trial was registered with the Clinical Research Information Service (CRIS) of the Republic of Korea (http://cris.nih.go.kr; registration no. KCT0009298) and was conducted in adherence to the principles of the Declaration of Helsinki, the International Council for Harmonisation (ICH) Good Clinical Practice (GCP) guidelines, and the Consolidated Standards of Reporting Trials (CONSORT) statement for randomized clinical trials. All participants provided written informed consent before enrollment.

### 2.2. Study Participants

A total of 100 participants with MCI were enrolled in this study. All participants were ethnically Korean. The inclusion criteria were: (1) men and women aged 55 to 80 years; (2) those whose Seoul Verbal Learning Test-Elderly’s version (SVLT-E) scores from the Seoul Neuropsychological Screening Battery-Second Edition (SNSB-II) were between 1.0 and 2.0 standard deviations (SD) below the mean of age- and education-matched controls; (3) those with the ability to read and comprehend the Korean; and (4) those who provided written informed consent voluntarily after receiving a comprehensive explanation of the trial.

Participants were excluded if they had conditions causing cognitive impairment, such as dementia, Parkinson’s disease, or attention deficit hyperactivity disorder, or if they had been prescribed medications known to impact cognitive functioning. In addition, individuals deemed by the investigator to be unsuitable for participation for other reasons were also excluded.

### 2.3. Study Design

A multicenter, randomized, double-blind, placebo-controlled clinical trial with a 12-week intervention period was conducted to evaluate the efficacy and safety of LL for enhancing cognitive function. The study was conducted at the Korean Medicine Hospitals of Wonkwang University (Jeonju, Republic of Korea) and Woosuk University between January and October 2024.

Dietary intake and physical activity were assessed at both baseline and the end of the trial. Participants were instructed to maintain their habitual dietary intake, regular physical activity, and overall food consumption throughout the study period. Changes in dietary habits were monitored using dietary records, which were analyzed by a registered dietitian utilizing the Computer-Aided Nutritional Analysis Program (CAN-pro; Korean Nutrition Society, Seoul, Republic of Korea).

**Figure 1:**
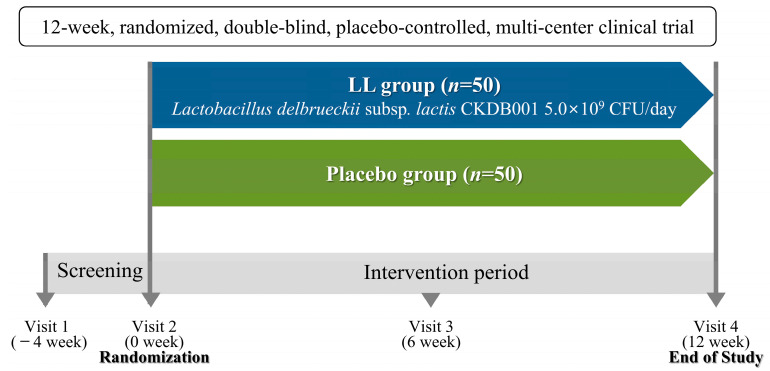
A schematic representation of the study design.

Participants attended their initial study visit within four weeks after the screening visit to determine eligibility based on the specified inclusion and exclusion criteria. In total, 100 eligible participants were randomly assigned in a 1:1 ratio to receive either LL (*n* = 50) or placebo (*n* = 50). All subjects attended four study visits: Visit 1 (week −4), Visit 2 (week 0), Visit 3 (week 6), and Visit 4 (week 12). Baseline data were collected at Visit 1 (week −4) or Visit 2 (week 0), and follow-up measurements were obtained at 6-week intervals during the subsequent visits. A schematic overview of the study design appears in Figure 1.

### 2.4. Study Products

LL (KCTC 14149BP), initially isolated from fermented milk, was provided by Chong Kun Dang Bio Corporation (Seoul, Republic of Korea). Its whole genome sequence is available in the National Center for Biotechnology Information (NCBI) database under the accession number CP145818. The probiotics powder was obtained by culturing the strain in an optimized edible medium using a fermenter, followed by concentration and lyophilization.

Participants in the LL group received a daily dosage of 5.0 × 10^9^ CFU of LL, administered as two capsules (300 mg each). The LL capsules contained LL as the active ingredient, with microcrystalline cellulose and magnesium stearate as excipients.

The placebo capsules were composed of the same excipients (microcrystalline cellulose and magnesium stearate) but did not contain LL. Both LL and placebo products were manufactured in GMP-certified facilities of Chong Kun Dang Bio Corporation (Seoul, Republic of Korea), ensuring identical appearance, weight, and packaging. Study products were dispensed to participants at each study visit in pre-labeled containers according to the randomization schedule.

### 2.5. Efficacy Outcome Measures

Efficacy endpoints were evaluated at baseline and at the end of the 12-week intervention period to assess changes attributable to the supplementation. The primary efficacy endpoint was defined as the change in the total score of the Alzheimer’s Disease Assessment Scale–Cognition 13 (ADAS-cog-13). Secondary efficacy endpoints included the ADAS-cog-13 memory sub-score, Digit Span Test (DST), the Trail Making Test (TMT), and Stroop test.

Additional evaluations included measurement of biochemical parameters, including cytokines [tumor necrosis factor-α (TNF-α), interleukin-1β (IL-1β), and interleukin-10 (IL-10)], brain-derived neurotrophic factor (BDNF), and urinary 8-hydroxy-2′-deoxyguanosine (8-OHdG).

### 2.6. Microbiome and Metabolite Analysis Methods

#### 2.6.1. Fecal Sample Collection

Fecal samples were collected at two time points: at baseline and after 12 weeks of intervention. Participants were provided with sterile plastic containers and stool collection kits (mediclin^®^ Stool Container, DM.Con 3040; DAIHAN Medical, Seoul, Republic of Korea), and the collected samples were stored in a deep freezer until analysis.

#### 2.6.2. Microbiome Analysis

Genomic DNA was extracted from fecal samples, and the V3–V4 region of the 16S rRNA gene was amplified using primers 341F and 805R. PCR amplification and sequencing were outsourced to the National Instrumentation Center for Environmental Management (NICEM; Seoul, Republic of Korea), where sequencing was performed using the Illumina MiSeq platform. Raw sequencing data were subsequently processed and analyzed in-house using QIIME2, with DADA2 employed for denoising. Microbial taxonomic profiling was performed using the SILVA database (version 2024.07) to determine microbial abundance at the phylum, family, and genus levels. For visualization and comparison, taxa were grouped as follows: the top 20 most abundant taxa at each taxonomic level were displayed individually, while those with lower abundance were grouped into an “Other” category. Unassigned or unclassified taxa were categorized as “Unclassified”. Differential abundance analysis was conducted primarily at the family and genus levels, with particular attention to beneficial taxa such as *Lactobacillaceae* and *Bifidobacteriaceae* (family level), and *Lactobacillus* and *Bifidobacterium* (genus level). Group differences were assessed using the Wilcoxon rank-sum test on normalized taxonomic profiles. A total of 82 participants were included in the microbiota analysis (LL group: 42; placebo group: 40).

#### 2.6.3. Metabolite Analysis

Metabolomic analysis of fecal samples was carried out to quantify short-chain fatty acids (SCFAs)_,_ and indole-derived metabolites. SCFA quantification was conducted at the Korea Research Institute of Biomedical Science (KRIBS) using a 7890A gas chromatography–mass spectrometry (GC–MS) system (Agilent, Palo Alto, CA, USA) equipped with a flame ionization detector and a DB-FATWAX Ultra Inert column (30 m × 0.32 mm × 0.25 μm; Agilent, Palo Alto, CA, USA). A total of 56 participants were included in the SCFA analysis (LL group: 30; placebo group: 26).

The analysis of fecal metabolites was conducted at Metamass (Seoul, Republic of Korea) using an Orbitrap Exploris™ 120 mass spectrometer (Thermo Fisher Scientific, Waltham, MA, USA) coupled with a Vanquish binary pump C UHPLC system. Chromatographic separation was performed on a Kinetex C18 column (100 × 2.1 mm, 1.7 μm; Phenomenex, Torrance, CA, USA), and quality control samples were included to ensure analytical stability. Fecal metabolite extraction and UHPLC–MS/MS-based untargeted metabolomics analysis were outsourced to a commercial service provider (Metamass, Seoul, Republic of Korea). UHPLC-MS/MS data were acquired with Xcalibur software (version 2.0.0; Thermo Fisher Scientific). Raw files were converted to ABF (*.abf) format using ABF Converter (Reifycs Inc., Tokyo, Japan). After conversion, retention time correction, peak detection, alignment, and normalization were performed using MS–DIAL software (Version 5.1.230912; RIKEN, Kanagawa, Japan). MS-DIAL settings: MS1 tolerance = 0.01 Da; MS2 tolerance = 0.025 Da; minimum peak height = 10,000; alignment to pooled-QC (RT tolerance = 0.05 min; MS1 tolerance = 0.015 Da); all other parameters were kept at default. The aligned data were exported to Excel files (Microsoft, Redmond, WA, USA). Based on a prior report, we focused on indole-derived metabolites [28]. Indole-derived metabolites were provisionally identified by comparing experimental accurate mass, MS/MS spectra and retention times against reference databases, including the Human Metabolome Database (HMDB; http://www.hmdb.ca), the NIST spectral library, and a curated in-house collection of authentic standards. Identification adhered to the Metabolomics Standards Initiative (MSI) guidelines [29], with most metabolites classified as tentative (Level 2). Statistical analyses were performed using Prism (version 10.6.0; GraphPad, La Jolla, CA, USA). Within-group changes from baseline to week 12 were tested with the Wilcoxon signed-rank test. Between-group differences were tested on change scores (Δ = week 12—baseline) using the Wilcoxon rank-sum test. The analysis of indole-derived metabolites included 35 participants (LL group: 18; placebo group: 17).

### 2.7. Safety Outcome Measures

Safety evaluations were performed throughout the study by monitoring all adverse events (AEs) and conducting laboratory tests, vital sign measurements, and physical examinations at baseline and after 12 weeks of intervention. Fasting blood samples were obtained following a 12 h overnight fast for hematological, biochemical, and urinary analyses.

Laboratory evaluations encompassed complete blood count (CBC) [red blood cell (RBC), white blood cell (WBC) with differential count (neutrophil, lymphocyte, monocyte, eosinophil, basophil), hemoglobin, hematocrit, platelet], biochemistry profiles [albumin, total protein, glucose, blood urea nitrogen (BUN), serum creatinine, uric acid, total bilirubin, aspartate aminotransferase (AST), alanine aminotransferase (ALT), alkaline phosphatase (ALP), phosphorus (P), calcium (Ca), sodium (Na), potassium (K), chloride (Cl), total cholesterol, low-density lipoprotein cholesterol (LDL-C), high-density lipoprotein cholesterol (HDL-C), triglycerides (TG)], and urinalysis parameters [specific gravity, pH, protein (albumin), glucose, bilirubin, ketones, WBC, blood (RBC)]. Vital signs, such as systolic blood pressure (SBP), diastolic blood pressure (DBP), pulse rate, and body temperature, were recorded at each visit.

### 2.8. Statistical Analysis

The sample size was estimated based on observed changes in ADAS-cog before and after intervention for both the LL and placebo groups. The calculation referenced a previously published study with a similar design [30]. To account for an estimated dropout rate of 25%, the total required sample size was calculated to be 100 participants, allocating 50 subjects to each group via 1:1 randomization.

All statistical analyses were performed using SAS^®^ software (Version 9.4, SAS Institute, Cary, NC, USA). Continuous variables were presented as mean ± SD, and categorical variables were described using frequencies and percentages. The efficacy analyses were primarily based on the Full Analysis Set (FAS), and safety assessments were conducted on the Safety Set (SS), which included all participants who ingested the investigational product at least once.

Within-group analyses utilized either the paired *t*-test or the Wilcoxon signed-rank test, depending on the normality of the data distribution. Between-group differences in changes from baseline were assessed using the two-sample *t*-test or the Wilcoxon rank-sum test, as appropriate based on the distribution of the data. In addition, an analysis of covariance (ANCOVA) was conducted with the baseline value as a covariate to adjust for potential confounding effects. A *p*-value of less than 0.05 was considered statistically significant.

## 3. Results

### 3.1. Subject Characteristics

**Figure 2:**
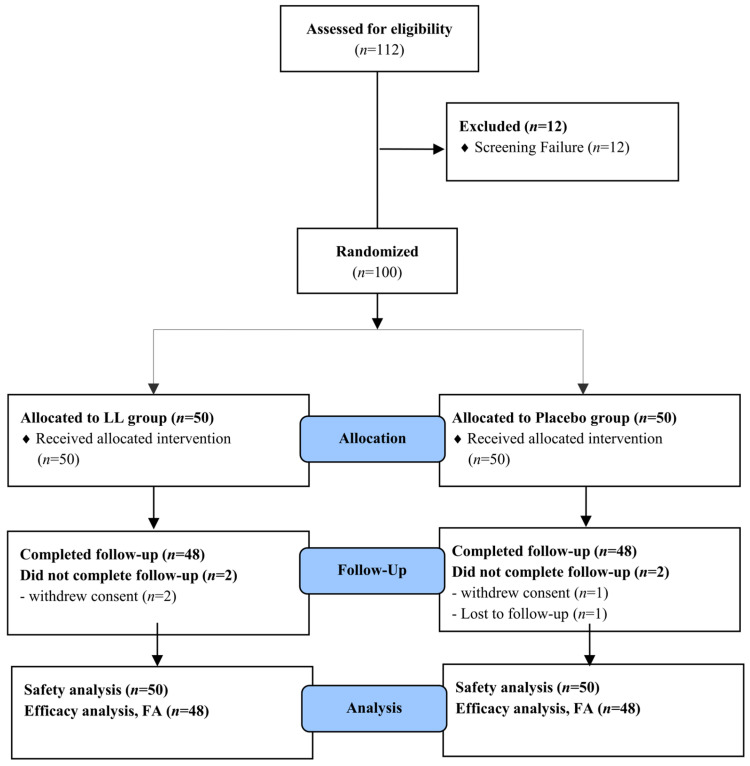
Flow-chart of subjects. The number of study participants enrolled, allocated, followed, and analyzed was shown using the CONSORT 2010 Flow Diagram.

Of the 112 volunteers screened, 100 subjects who fulfilled all inclusion and exclusion criteria were randomized to either the LL or placebo group. Four subjects with incomplete primary efficacy data were excluded from the FAS. As a result, a total of 96 participants (48 per group) were included in the FAS and completed all study-related procedures in accordance with the study protocol (Figure 2).

Analysis of intake compliance showed that no participants were withdrawn due to insufficient adherence. The mean compliance rate was 95.57 ± 5.87% in the LL group and 95.81 ± 4.76% in the placebo group, with no statistically significant difference between groups (*p* > 0.05).

Table 1 presents the demographic characteristics of all participants. The study population consisted of 22 males and 74 females. The mean age for the LL group was 67.33 ± 4.61 years, while placebo group had a mean age of 67.35 ± 4.96 years; no statistically significant difference was observed between the two groups (*p* > 0.05).

No significant differences were observed between the groups in terms of education level, height, weight. Similarly, baseline ADAS-cog-13 total scores, representing the primary efficacy endpoint, did not differ significantly between the groups. Collectively, these findings indicate that baseline characteristics were well-matched, reducing the likelihood that initial group, differences influenced study outcomes.

### 3.2. Efficacy Evaluation

**Figure 3:**
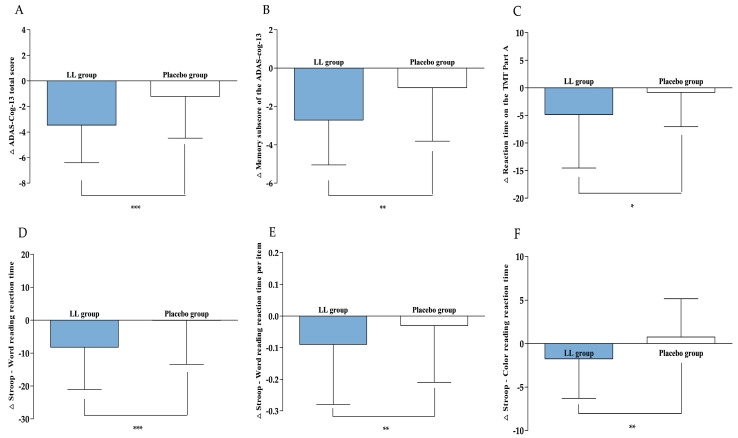
Changes in efficacy outcomes. (**A**) ADAS-Cog-13 total score, (**B**) Memory sub-score of the ADAS-cog-13, (**C**) Reaction time on the TMT Part A, (**D**) Stroop—Word reading reaction time, (**E**) Stroop—Word reading reaction time per item, and (**F**) Stroop—Color reading reaction time were measured in LL and placebo groups at baseline and 12 weeks. Values are presented as mean ± SD. Analyzed by Wilcoxon rank sum test between the groups at change value. Statistical significance was defined as *p* < 0.05, *p* < 0.01, and *p* < 0.001, and significant differences are indicated by asterisks (*, **, ***).

Neurocognitive function was evaluated using efficacy endpoints, including ADAS-cog-13, DST, TMT, and the Stroop test, administered both at baseline and after 12 weeks of intervention. The results are provided in Table 2 and Figure 3.

After 12 weeks of intervention, the ADAS-cog-13 total score, serving as the primary efficacy endpoint, significantly decreased in both groups compared to baseline (LL group: −3.46 ± 2.95, *p* < 0.0001; placebo group: −1.21 ± 3.27, *p* = 0.0077). The reduction was greater in the LL group, resulting in a statistically significant difference between the groups (*p* = 0.0003). An ANCOVA, with baseline values as covariates, further verified that the decrease was significantly greater in the LL group versus the placebo group (*p* < 0.0001), confirming the consistency of this trend.

Regarding the secondary efficacy endpoint, change in the memory sub-score of the ADAS-cog-13 were −2.71 ± 2.34 for the LL group and −1.02 ± 2.79 for the placebo group. Both groups demonstrated significant within-group reductions (LL group: *p* < 0.0001; placebo group: *p* = 0.0148). The LL group achieved a more pronounced reduction, leading to a significant between-group difference (*p* = 0.0044). ANCOVA, adjusted for baseline values, corroborated these results, indicating a significantly greater decrease in the LL group (*p* < 0.0001) and supporting the robustness of the statistical findings.

In this study, the TMT was administered at baseline and again after 12 weeks of intervention. The LL group demonstrated a change in reaction time on the TMT Part A of −4.83 ± 9.71 s, whereas the placebo group exhibited a change of −0.81 ± 6.24 s. A statistically significant reduction in reaction time occurred within the LL group (*p* < 0.0001), while the placebo group did not show a significant difference (*p* = 0.3712). Additionally, between-group analysis identified a significant difference favoring the LL group (*p* = 0.0167). Regarding the number of errors, the LL group showed a change of −0.04 ± 0.41, and the placebo group had an increase of +0.10 ± 0.31, suggesting a minor decrease in errors for the LL group as opposed to an increase in the placebo group. A trend toward a between-group difference was observed (*p* = 0.0600). However, this did not indicate statistical significance. ANCOVA adjusted for baseline values verified that the reduction in reaction time was significantly greater in the LL group compared to the placebo group (*p* = 0.0129), whereas no significant group difference was detected for the number of errors (*p* > 0.05).

In this study, Stroop test performance changes from baseline to week 12 were assessed for both word-reading and color-reading tasks. The reaction time in the word-reading task changed by −8.23 ± 12.85 s in the LL group and −0.06 ± 13.38 s in the placebo group, with a significant reduction present only in the LL group (*p* < 0.0001 versus *p* = 0.1867, respectively). Statistical comparison between groups showed a significant difference (*p* < 0.0001). Baseline-adjusted ANCOVA analyses supported these observations (*p* = 0.0003). For mean reaction time per item in word reading, changes measured were −0.09 ± 0.19 s in the LL group and −0.03 ± 0.18 s in the placebo group; only the LL group experienced a significant reduction (*p* < 0.0001), while the placebo group did not (*p* = 0.5003). The difference between groups was also statistically significant (*p* = 0.0011), and baseline-adjusted ANCOVA confirmed this pattern (*p* = 0.0018). In the color-reading task, the LL group had a reaction time change of −1.75 ± 4.55 s, compared to 0.75 ± 4.42 s in the placebo group. There was a significant decrease in the LL group (*p* = 0.0066), with no notable change in the placebo group (*p* = 0.1875). Between-group analysis further confirmed a significant difference (*p* = 0.0026), and the findings remained consistent when analyzed using baseline-adjusted ANCOVA (*p* = 0.0111). Other measured parameters did not show significant differences between groups.

The biochemical parameters assessed, including cytokines (TNF-α, IL-1β, IL-10), BDNF, and urinary 8-OHdG, were evaluated at baseline and after 12 weeks of intervention, as summarized in Table 3. Within the placebo group, the pro-inflammatory cytokine TNF-α demonstrated a statistically significant increase (*p* = 0.0032), while the LL group showed no significant change (*p* = 0.5361). A baseline-adjusted ANCOVA analysis indicated a trend toward a between-group difference (*p* = 0.0678). However, this did not reach statistical significance (*p* > 0.05). Similarly, a significant increase in the pro-inflammatory cytokine IL-1β was observed within the placebo group (*p* = 0.0014), but not in the LL group (*p* = 0.2097), and no significant difference was found between groups (*p* > 0.05). No other parameters demonstrated significant differences between the intervention groups.

### 3.3. Results of Microbiome and Metabolite Analysis

**Figure 4:**
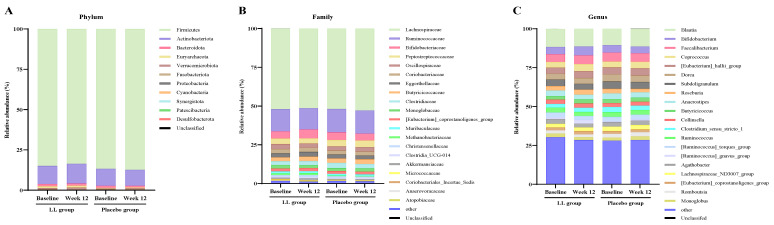
Relative abundance of gut microbiota at different taxonomic levels in fecal samples from the LL and placebo groups. Stacked bar plots depict the average relative abundance of gut microbiota at the (**A**) phylum, (**B**) family, and (**C**) genus levels. Fecal samples were collected from the LL (*n* = 42) and placebo (*n* = 40) groups at baseline and after 12 weeks of intervention. Taxonomic profiling was performed using QIIME2 and the SILVA database (version 2024.07). The top 20 most abundant taxa at each taxonomic level are shown individually; remaining low-abundance taxa were grouped as “Other,” and unclassified taxa are labeled as “Unclassified”.

The composition of the gut microbiota was profiled at the phylum, family, and genus levels using fecal samples (Figure 4). At week 12, the LL group showed an increased abundance of Actinobacteria (phylum), which includes *Bifidobacteria*. Consistently, increases in *Bifidobacteriaceae* (family) and *Bifidobacterium* (genus) were also observed in the same group. Several genera associated with cognitive function and butyrate production showed favorable changes in their relative abundance. *Faecalibacterium* increased in the LL group from 4.97% to 5.56%, while it slightly declined in the placebo group from 5.74% to 5.45%. The *Eubacterium hallii* group rose from 3.97% to 4.47% in the LL group and from 4.45% to 4.70% in the placebo group. *Roseburia* also showed a notable increase in the LL group (2.80% to 3.26%) but decreased markedly in the placebo group (2.90% to 2.19%). *Butyrivibrio*, which was undetectable at baseline in the LL group, appeared after the intervention (0.10%), whereas it slightly declined in the placebo group (0.03% to 0.02%). However, despite these compositional changes, within-group and between-group comparisons for these genera did not reach statistical significance. In contrast, significant differences were observed in *Bifidobacteriaceae* and *Lactobacillaceae* (family level), as well as *Bifidobacterium* and *Lactobacillus* (genus level), which are well-recognized beneficial gut microbes.

**Figure 5:**
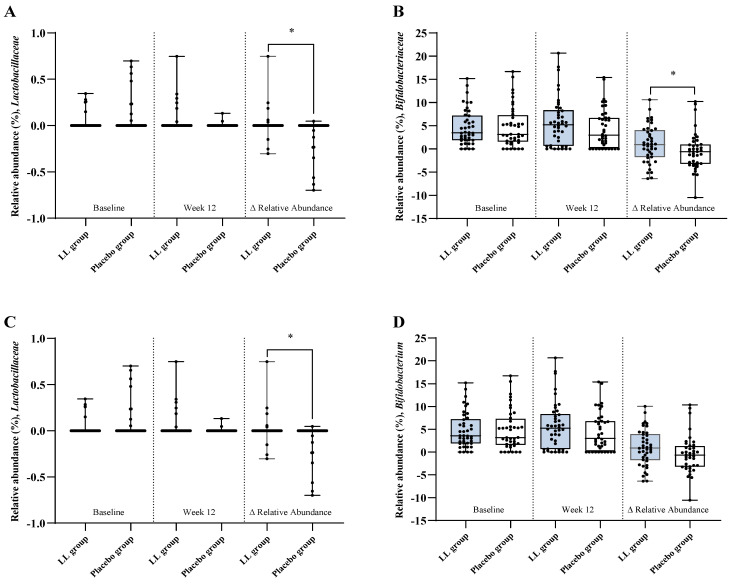
Relative abundance of gut microbial taxa. Taxonomic profiling of fecal samples collected at baseline and week 12 was conducted to evaluate the relative abundance of specific gut microbial taxa in the LL and placebo groups. Panels show results for (**A**) *Lactobacillaceae* and (**B**) *Bifidobacteriaceae* at the family level, and (**C**) *Lactobacillus* and (**D**) *Bifidobacterium* at the genus level. Each panel displays group comparisons at baseline (left), week 12 (middle), and the change from baseline to week 12 (Δ; right). Values are presented as mean ± SD. Between-group comparisons at each time point and for Δ relative abundance (Week 12—Baseline) were performed using the Wilcoxon rank-sum test. Statistical significance was defined as *p* < 0.05, and significant differences are indicated by an asterisk (*).

Changes in the relative abundance of gut microbiota from baseline to week 12 are presented in Figure 5. The LL group exhibited a significantly greater increase in the relative abundance of *Lactobacillaceae* (*p* = 0.0247) and *Bifidobacteriaceae* (*p* = 0.0436) at the family level compared with the placebo group. At the genus level, the LL group demonstrated a significantly greater increase in the relative abundance of *Lactobacillus* (*p* = 0.0247). *Bifidobacterium* showed a non-significant trend toward a greater increase in the LL group compared with the placebo group (*p* = 0.0554).

Metabolomic analyses were conducted at baseline and after 12 weeks of intervention, with the findings presented in Table 4. Of the SCFAs measured, butyric acid showed a statistically significant increase from baseline to week 12 within the LL group (*p* = 0.0449), while acetic acid and propionic acid did not exhibit significant within-group changes. Nonetheless, no significant between-group differences in changes from baseline to week 12 were observed for any of the SCFAs. Assessment of indole-derived metabolites revealed that, for 5-HIAA, the LL group had a relative increase of 1.04 × 10^−2^ ± 3.45 × 10^−2^ peak area from baseline, in contrast to a decrease of −1.88 × 10^−2^ ± 4.98 × 10^−2^ peak area in the placebo group, yielding a statistically significant between-group difference (*p* = 0.0224). Similarly, ILA showed a relative increase of 0.93 × 10^−3^ ± 5.44 × 10^−3^ peak area in the LL group, whereas the placebo group exhibited a decrease of −4.83 × 10^−3^ ± 18.10 × 10^−3^ peak area, and this between-group difference achieved statistical significance (*p* = 0.0209). In the case of indole-3-glycol, the LL group demonstrated a relative increase of 0.81 × 10^−3^ ± 2.58 × 10^−3^ peak area, while the placebo group had only a minimal change (0.09 × 10^−3^ ± 2.80 × 10^−3^ peak area), with the between-group comparison also revealing a significant difference (*p* = 0.0487). No statistically significant differences between groups were observed in the remaining parameters.

### 3.4. Safety Evaluation

Safety assessments were conducted in the safety analysis set. During the intervention period, a single AE of nasopharyngitis was documented in the LL group, and no AEs were reported in the placebo group. The incidence did not differ significantly between groups (*p* > 0.05), and the reported AE was assessed as unrelated to the study product. No serious AEs were observed in either group.

Across the 12 week period, no statistically significant group differences were detected in laboratory parameters such as complete blood count, serum biochemistry, or urinalysis. All laboratory profiles are presented in Supplementary Table S1. Furthermore, other safety outcomes, including vital signs and findings on physical examination also showed no significant group differences. Collectively, these findings indicate that the study product demonstrated a favorable safety and tolerability profile.

## 4. Discussion

In this study, we performed a randomized, double-blind, placebo-controlled, multicenter trial to determine the efficacy and safety of LL in individuals with MCI. To our knowledge, this represents the first study to investigate the effects of LL on both cognitive function and gut microbiota in this population. After 12 weeks, LL supplementation resulted in significant improvements versus the placebo group in the ADAS-cog-13 total score, the memory sub-score, TMT Part A reaction time, and word- and color-reading reaction times on the Stroop test. In addition, LL supplementation was associated with beneficial changes in gut microbiota, increased production of indole-derived metabolites, and a favorable safety profile.

Participants in this trial were older adults aged 55–80 years, with a mean age of 67.33 ± 4.61 years in the LL group and 67.35 ± 4.96 years in the placebo group. This age bracket has been identified as well-suited for interventions that aim to maintain cognitive function prior to the development of advanced frailty or dementia [6]. By targeting individuals experiencing early cognitive decline, this investigation is more consistent with an early intervention strategy rather than primary prevention. This chosen study design allows for assessment of LL’s effects during a period when alteration of cognitive decline trajectories may remain feasible [6].

ADAS-Cog is among the most commonly utilized tools for assessing cognitive function within clinical trials and is recognized as a gold standard for evaluating the efficacy of interventions aimed at dementia and cognitive decline [31,32]. It demonstrates particular sensitivity in detecting treatment effects in individuals with MCI or early dementia [33]. In the ADAS-Cog-13, higher scores reflect increased severity of cognitive impairment [34]. In this study, LL supplementation for 12 weeks led to significant improvement in the ADAS-Cog-13 total score as well as the memory sub-score when compared to the placebo group, suggesting not only overall cognitive benefits but also benefit as well as specific enhancement in memory function. Interestingly, significant within-group improvements were also observed in some cognitive measures in the placebo group, such as the ADAS-cog-13 and memory sub-scores. This phenomenon is likely attributable to practice effects, such as increased familiarity with repeated test administration, which are well documented in neuropsychological assessments of older adults [35,36,37]. However, these improvements were modest compared with those observed in the LL group, suggesting that the between-group differences reflect a genuine intervention effect rather than mere retest familiarity. These results align with multiple clinical trials indicating that probiotic supplementation may enhance cognitive performance in individuals with MCI or early Alzheimer’s disease, particularly impacting memory and overall cognition [6,38]. Conversely, previous research did not observe significant changes in ADAS-Cog scores following probiotic interventions [39], indicating that variables such as strain specificity, duration of intervention, baseline cognitive function, and study design may impact the outcomes. Collectively, the improvements in ADAS-Cog scores observed in this trial highlight the potential of LL as a targeted probiotic approach for improving global cognition and memory among older adults with MCI.

Probiotics are live microorganisms that, when administered at sufficient dosages, offer health benefits to the host, including potential impacts on neural health through modulation of the gut–brain axis [23,40,41,42,43]. There is growing evidence that probiotics may affect cognitive functioning by altering gut microbiota profiles, influencing neuroactive metabolite production, and modulating immune as well as inflammatory responses [44]. Multiple clinical investigations have shown that probiotic supplementation can yield cognitive advantages in individual with MCI and early-stage dementia, specifically improving domains such as memory, attention, and executive function [6,9,38,45,46,47,48,49,50,51,52,53,54]. Consistent with these reports, the cognitive improvements documented in our current study reinforce the proposition that targeted probiotic interventions may offer a feasible approach to mitigating cognitive decline in elderly populations susceptible to developing dementia.

The DST is a widely used neuropsychological measure of short-term memory, working memory, and attention [55]. In the present study, no significant changes were observed in DST performance in either group after 12 weeks of intervention. Previous clinical trials on probiotic supplementation in individuals with MCI have reported inconsistent effects on working memory and attention, with some studies showing modest improvements and others finding no significant changes [56]. These findings, together with our results, suggest that LL supplementation may exert greater effects on cognitive domains related to processing speed, executive function, and inhibitory control, rather than on basic attention span or short-term memory capacity.

The TMT is a well-established neuropsychological assessment that measures executive function, processing speed, attention, and visuospatial scanning ability [57,58]. In our study, a significant reduction in reaction time on TMT Part A was observed in the LL group compared to the placebo group, whereas error counts showed a trend toward improvement without reaching statistical significance. Similar improvements in TMT performance have been reported in interventions targeting processing speed and executive function in populations with MCI [59,60], supporting the notion that LL supplementation may enhance neural efficiency and cognitive flexibility in these domains.

The Stroop test is a neuropsychological assessment designed to evaluate attention, processing speed, executive function, and inhibitory control [61,62]. In this study, LL supplementation resulted in significant improvements in both word-reading and color-reading tasks, including faster reaction times and improved per-item response speed, compared with the placebo group. These findings are in line with previous studies demonstrating that probiotic or dietary interventions can improve inhibitory control and processing speed in older adults at risk of cognitive decline [38,49]. Collectively, the improvements in TMT and Stroop performance observed in our study support the potential of LL supplementation to target higher-order cognitive functions that are critical for daily functioning in at-risk older adults.

Beyond cognitive outcomes, LL supplementation for 12 weeks resulted in a significantly greater change from baseline in the relative abundance of beneficial taxa such as *Lactobacillaceae*, *Bifidobacteriaceae*, and *Lactobacillus* in the LL group compared to the placebo group. These findings are consistent with previous reports demonstrating that probiotic supplementation promotes the growth of beneficial microbes [63,64], supporting the possibility that modulation of the gut environment may contribute to cognitive improvement. SCFAs are key metabolites produced by beneficial gut microbiota and play a critical role in maintaining the gut–brain axis. Among them, butyric acid is particularly notable for its ability to regulate neuroinflammation and enhance cognitive function through the inhibition of histone deacetylase (HDAC) [65,66]. In this study, butyric acid levels significantly increased in the LL group following LL supplementation (*p* = 0.0449), whereas a decreasing trend was observed in the placebo group, suggesting that LL may promote the production of beneficial gut-derived metabolites. Although the taxonomic shifts in butyrate-producing genera did not reach statistical significance, the relative abundance of genera such as *Faecalibacterium*, *Eubacterium hallii*, *Roseburia*, and *Butyrivibrio* showed an increasing trend in the LL group. Most of these genera are well-known butyrate producers, and some of them, particularly *Faecalibacterium* and *Roseburia*, have been associated with improved cognitive outcomes in individuals with cognitive impairment or neurodegenerative conditions [67,68,69,70,71,72]. Thus, the observed increase in butyric acid levels may reflect functionally meaningful changes in the gut microbiota, even in the absence of statistically significant alterations in specific taxa.

Among probiotics, the genus *Lactobacillus*, comprising Gram-positive, thermophilic bacteria, has garnered significant interest due toattracted considerable attention for its economic significance in various industrial sectors [25]. Of the 42 recognized species of *Lactobacillus delbrueckii*, the subspecies *lactis* plays a pivotal role in the commercial manufacture of fermented products, notably yogurt and cheese [73]. Multiple investigations have established that *L. delbrueckii* subsp. *lactis* CKDB001 (LL) is a safe and efficacious probiotic with potential therapeutic benefits for metabolic dysfunction–associated steatotic liver disease (MASLD) [25,26]. Preclinical studies involving LL have indicated its capability to modulate key biological pathways relevant to metabolic and neurodegenerative diseases [27,28]. In a high-fat diet-induced obese mouse model [27], supplementation with LL reorganized the gut microbial ecosystem, increasing beneficial bacterial populations such as *Lactobacillus* spp. while decreasing pro-inflammatory bacteria, including *Alistipes* and *Bilophila*. Functional predictions suggested suppression of lipopolysaccharide (LPS) and ADP-L-glycero-β-D-manno-heptose biosynthesis, alongside enhancement of carbohydrate metabolism and SCFA production pathways. Such shifts in microbial composition and function may attenuate peripheral endotoxemia and lower systemic inflammatory tone, thereby positively influencing gut–brain axis signaling. Cognitive outcomes have also been explored in the 5xFAD transgenic mouse model of Alzheimer’s disease [28], where LL administration significantly reduced cortical soluble Aβ42 levels. This effect has been linked to the microbial metabolite ILA, a tryptophan-derived compound that activates AhR in microglia and astrocytes. AhR activation was associated with enhanced Aβ phagocytosis, suppression of NF-κB–mediated inflammatory signaling, and decreased amyloid accumulation. Moreover, co-administration of tryptophan and ILA further decreased both soluble and insoluble Aβ species and preserved memory performance in behavioral assays. In the present clinical trial, LL supplementation led to significant improvements in certain cognitive performance measures compared with placebo, alongside an observed increase in *Lactobacillus* spp. abundance within the gut microbiota. These findings are consistent with mechanistic evidence from non-clinical studies, suggesting that LL may contribute to cognitive improvement through modulation of gut microbiota, enhancement of anti-inflammatory and anti-amyloid metabolite production, and regulation of neuroinflammation. Collectively, LL appears to be a promising functional ingredient for maintaining cognitive health by simultaneously improving gut environment and providing neuroprotection.

In addition to microbial shifts, LL supplementation led to significant increases in indole-derived metabolites (5-HIAA, ILA, and indole-3-glycol), which have been shown to activate the aryl hydrocarbon receptor (AhR) pathway, attenuate neuroinflammation, and suppress amyloid pathology, thereby potentially enhancing cognitive function [28,74]. These results suggest that LL may contribute to cognitive improvement by modulating the gut environment and enhancing the production of neuroactive metabolites through the gut–brain axis. Taken together, this supports the idea that targeting the gut–brain axis via specific probiotic interventions may represent a promising approach for maintaining and improving cognitive health in individuals with MCI.

In the present study, TNF-α and IL-1β levels showed significant increases over 12 weeks in the placebo group, whereas no significant changes were observed in the LL group. Although between-group comparisons did not show statistical significance, baseline-adjusted ANCOVA revealed a borderline significance for TNF-α, suggesting a potential anti-inflammatory effect of LL supplementation. In contrast, no significant differences between groups were found for IL-10, BDNF, or urinary 8-OHdG. These biochemical biomarkers can be influenced by various factors, including individual health status, lifestyle, and age, which may partly explain the discrepancies from results observed in animal studies [75,76,77,78]. Thus, the potential anti-inflammatory properties of LL may represent an important mechanistic pathway through which it could modulate the pathophysiology of cognitive decline, warranting further long-term and large-scale investigations.

In terms of safety, only one adverse event was reported during the 12-week intervention, which was deemed unrelated to the study product. No clinically significant changes were observed in laboratory parameters or vital signs. These findings support the tolerability of LL supplementation and suggest its potential safety for longer-term use.

Randomized controlled trials (RCTs) present multiple methodological challenges; nevertheless, they remain the gold standard for producing reliable data in clinical research [53]. In studies involving older adults, ensuring ongoing follow-up and consistent participant contact is particularly demanding, often leading to higher dropout rates and lower compliance [79,80]. Despite these inherent difficulties, the current study attained a compliance rate above 95% and a dropout rate limited to 4%. These favorable outcomes were most likely achieved through rigorous study oversight, proactive participant engagement, and the relatively brief duration of the intervention period. Additionally, recent research has demonstrated a significant association between intervention compliance and cognitive improvement [50,81]. Therefore, the cognitive enhancements observed in our trial can plausibly be partly ascribed to the high compliance levels and low dropout rates, which together enhance the robustness of our study conclusions.

This study has several limitations that should be considered when interpreting the findings. First, the intervention period was relatively short (12 weeks), which may not be sufficient to fully capture the long-term effects of LL supplementation on cognitive decline. Nonetheless, numerous previous studies in individuals with MCI have reported cognitive improvements following dietary supplementation periods of ≤12 weeks [82,83,84], indicating that a 12-week intervention paradigm is widely implemented to assess the cognitive benefits of nutritional supplementation. Second, although the present study had an adequate sample size to detect significant differences in primary outcomes, larger-scale studies are warranted to validate these results and explore subgroup effects, such as differences by sex, baseline cognitive status, and gut microbiota composition. Notably, there was an imbalance in sex distribution, with a predominance of female participants. This likely reflects the higher prevalence of MCI among older women, as reported in previous epidemiological studies [85,86]. At baseline, there was no statistically significant difference in sex distribution between the groups (*p* = 0.3314); therefore, sex was not included as a covariate in the ANCOVA model. Nevertheless, this imbalance should be considered when interpreting the findings, and future studies with more balanced recruitment are needed to clarify potential sex-specific effects. Third, while multiple cognitive domains and gut microbiota profiles were assessed, other potential mechanisms, including neuroimaging biomarkers and neuroinflammatory pathways, were not examined. Fourth, the study population was restricted to older adults with MCI, which limits the generalizability of the findings to other populations, such as those with more advanced dementia or younger individuals at risk of cognitive decline. Therefore, future research should involve longer intervention periods, more diverse populations, and broader mechanistic assessments to validate and extend the current findings.

Despite these limitations, this study possesses several strengths, including a robust RCT design, very high compliance (>95%), minimal dropout (4%), and an integrated assessment spanning cognition, gut microbiota, and biochemical markers in older adults with MCI.

## 5. Conclusions

This randomized controlled trial represents the first evaluation of the effects of *Lactobacillus delbrueckii* subsp. *lactis* CKDB001 (LL) supplementation in MCI. Over the 12-week period, LL supplementation led to improvements in overall cognitive function, offered mechanistic evidence through beneficial changes in gut microbiota and metabolite profiles, and was found to be safe and well-tolerated. Collectively, these results provide robust clinical evidence for LL as a health functional food to enhance cognitive function.

## Figures and Tables

**Table 1:** Baseline demographic characteristics of subjects.

	LL Group (*n* = 48)	Placebo Group (*n* = 48)	*p*-Value ^1^
Sex (M/F)	9/39	13/35	0.3314 ^2^
Age (years)	67.33 ± 4.61	67.35 ± 4.96	0.9941 ^3^
Education (years)	11.65 ± 3.06	11.14 ± 3.38	0.4720 ^3^
Height (cm)	158.23 ± 6.93	158.55 ± 8.41	0.8399
Weight (kg)	60.18 ± 9.08	61.05 ± 8.85	0.6362
ADAS-cog-13 total score	17.77 ± 4.85	18.50 ± 4.37	0.4411

Values are presented as mean ± SD or number (%). ^1^ Analyzed by two sample *t*-test between the groups. ^2^ Analyzed by chi-square test between the groups. ^3^ Analyzed by Wilcoxon rank sum test between the groups.

**Table 2:** Changes in neurocognitive function tests before and after 12 weeks of intake.

			LL Group (*n* = 48)				Placebo Group (*n* = 48)				*p*-Value ^3^	*Adj.p*-Value ^4^
			Baseline	12 Week	Change Value	*p*-Value ^1^	Baseline	12 Week	Change Value	*p*-Value ^1^		
ADAS-cog-13	Total score		17.77 ± 4.85	14.31 ± 3.88	−3.46 ± 2.95	<0.0001 ^2^	18.50 ± 4.37	17.29 ± 4.05	−1.21 ± 3.27	0.0077 ^2^	0.0003	<0.0001
	Memory sub-score		12.65 ± 3.55	9.94 ± 2.87	−2.71 ± 2.34	<0.0001 ^2^	13.38 ± 3.37	12.35 ± 3.29	−1.02 ± 2.79	0.0148	0.0044	<0.0001
DST	Digit span Forward		6.13 ± 1.27	6.19 ± 0.94	0.06 ± 1.00	0.6927 ^2^	5.60 ± 1.16	5.65 ± 1.04	0.04 ± 0.82	0.8274 ^2^	0.7725	0.0999
	Digit span Backward		3.75 ± 0.84	3.94 ± 1.00	0.19 ± 1.04	0.2154 ^2^	3.56 ± 0.80	3.56 ± 0.80	0.00 ± 0.85	1.0000 ^2^	0.3481	0.0900
	DF-DB		2.38 ± 1.06	2.25 ± 1.06	−0.13 ± 1.28	0.6094 ^2^	2.04 ± 1.24	2.08 ± 1.01	0.04 ± 1.25	0.7762 ^2^	0.5322	0.7405
TMT	Part A	Reaction time	30.54 ± 11.10	25.71 ± 9.13	−4.83 ± 9.71	<0.0001 ^2^	30.02 ± 10.02	29.21 ± 11.33	−0.81 ± 6.24	0.3712	0.0167	0.0129
		Number of errors	0.10 ± 0.37	0.06 ± 0.24	−0.04 ± 0.41	0.7500 ^2^	0.02 ± 0.14	0.13 ± 0.33	0.10 ± 0.31	0.0625 ^2^	0.0600	0.1824
	Part B	Reaction time	50.13 ± 27.22	44.67 ± 23.54	−5.46 ± 21.55	0.0095 ^2^	57.69 ± 31.99	54.06 ± 32.83	−3.63 ± 32.06	0.0809 ^2^	0.5849	0.2801
		Number of errors	0.50 ± 1.07	0.50 ± 0.83	0.00 ± 1.35	0.8073 ^2^	0.98 ± 1.39	0.71 ± 1.15	−0.27 ± 1.76	0.3288 ^2^	0.405	0.3503
Stroop test	Word reading	Reaction time	81.73 ± 17.55	73.50 ± 12.83	−8.23 ± 12.85	<0.0001 ^2^	81.73 ± 19.19	81.67 ± 17.19	−0.06 ± 13.38	0.1867 ^2^	<0.0001	0.0003
		Reaction time per item	0.75 ± 0.23	0.66 ± 0.11	−0.09 ± 0.19	<0.0001 ^2^	0.77 ± 0.26	0.74 ± 0.18	−0.03 ± 0.18	0.5003 ^2^	0.0011	0.0018
		Number of correct responses	109.58 ± 7.65	111.60 ± 0.96	2.02 ± 7.60	<0.0001 ^2^	108.04 ± 8.60	110.85 ± 3.96	2.81 ± 7.71	0.0003 ^2^	0.7470	0.3146
		Number of errors	1.13 ± 1.62	0.40 ± 0.96	−0.73 ± 1.22	<0.0001 ^2^	0.98 ± 1.21	0.58 ± 1.07	−0.40 ± 1.25	0.0331 ^2^	0.3032	0.1716
		Correct response rate	0.99 ± 0.02	1.00 ± 0.01	0.01 ± 0.01	<0.0001 ^2^	0.99 ± 0.01	0.99 ± 0.01	0.00 ± 0.01	0.0143 ^2^	0.3260	0.2137
	Color reading	Reaction time	118.67 ± 4.44	116.92 ± 6.61	−1.75 ± 4.55	0.0066 ^2^	117.29 ± 8.85	118.04 ± 5.31	0.75 ± 4.42	0.1875 ^2^	0.0026	0.0111
		Reaction time per item	1.42 ± 0.45	1.30 ± 0.37	−0.12 ± 0.23	<0.0001 ^2^	1.47 ± 0.44	1.36 ± 0.44	−0.10 ± 0.30	0.0229 ^2^	0.3911	0.6164
		Number of correct responses	87.10 ± 19.36	93.15 ± 17.83	6.04 ± 11.29	<0.0001 ^2^	82.81 ± 19.66	90.06 ± 18.43	7.25 ± 12.98	<0.0001 ^2^	0.8141	0.9713
		Number of errors	2.21 ± 2.17	1.40 ± 1.73	−0.81 ± 1.96	0.0071 ^2^	2.48 ± 3.24	1.88 ± 2.42	−0.60 ± 2.16	0.0952 ^2^	0.6173	0.2920
		Correct response rate	0.97 ± 0.03	0.98 ± 0.02	0.01 ± 0.03	0.0034 ^2^	0.97 ± 0.05	0.98 ± 0.03	0.01 ± 0.03	0.0204 ^2^	0.7856	0.3184
	Stroop interference		0.67 ± 0.44	0.65 ± 0.34	−0.02 ± 0.27	0.7621 ^2^	0.70 ± 0.36	0.63 ± 0.37	−0.07 ± 0.31	0.0447 ^2^	0.2468	0.4556

Values are presented as mean ± SD. ^1^ Analyzed by paired *t*-test between baseline and 12 weeks within each group. ^2^ Analyzed by Wilcoxon signed rank test between baseline and 12 weeks within each group. ^3^ Analyzed by Wilcoxon rank sum test between the groups at change value. ^4^ Analyzed by ANCOVA adjusted baseline.

**Table 3:** Changes in cytokine, BDNF, and 8-OHdG levels before and after 12 weeks of intake.

		LL Group (*n* = 48)				Placebo Group (*n* = 48)				*p*-Value ^3^	*Adj.p*-Value ^4^
		Baseline	12 Week	Change Value	*p*-Value ^1^	Baseline	12 Week	Change Value	*p*-Value ^1^		
Cytokine (pg/mL)	TNF-α	0.57 ± 0.16	0.57 ± 0.14	0.01 ± 0.09	0.5361	0.56 ± 0.14	0.61 ± 0.18	0.05 ± 0.13	0.0032 ^2^	0.1893	0.0678
	IL-1β	0.07 ± 0.02	0.07 ± 0.03	0.00 ± 0.03	0.2097 ^2^	0.07 ± 0.03	0.08 ± 0.05	0.01 ± 0.04	0.0014 ^2^	0.1320	0.2671
	IL-10	0.88 ± 0.31	0.91 ± 0.33	0.02 ± 0.25	0.5434	0.83 ± 0.21	0.87 ± 0.27	0.04 ± 0.20	0.1626 ^2^	0.8231	0.9451
BDNF (pg/mL)		22,497.92 ± 9798.25	25,112.50 ± 9128.32	2614.58 ± 10,323.82	0.0251 ^2^	21,166.67 ± 8937.95	24,010.42 ± 9703.70	2843.75 ± 9250.78	0.0385	0.8575	0.7761
Urine 8-OHdG (mg/mL)		5.38 ± 4.52	8.05 ± 5.04	2.67 ± 5.63	0.0006 ^2^	3.88 ± 3.32	9.11 ± 8.55	5.22 ± 7.74	<0.0001 ^2^	0.0553	0.1565

Values are presented as mean ± SD. ^1^ Analyzed by paired *t*-test between baseline and 12 weeks within each group. ^2^ Analyzed by Wilcoxon signed rank test between baseline and 12 weeks within each group. ^3^ Analyzed by Wilcoxon rank sum test between the groups at change value. ^4^ Analyzed by ANCOVA adjusted baseline.

**Table 4:** Changes in metabolomics before and after 12 weeks of intake.

		LL Group (*n* = 30)				Placebo Group (*n* = 26)				*p*-Value ^2^
		Baseline	12 Week	Change Value	*p*-Value ^1^	Baseline	12 Week	Change Value	*p*-Value ^1^	
SCFA (µmol/g)	Acetic Acid	27.71 ± 20.70	24.92 ± 23.21	−2.782 ± 30.18	0.8078	29.35 ± 16.32	30.04 ± 24.38	0.686 ± 27.79	0.7979	0.9902
	Propionic Acid	20.64 ± 15.97	17.30 ± 18.30	−3.343 ± 22.81	0.4645	22.62 ± 14.79	22.60 ± 22.65	−0.011 ± −20.89	0.9205	0.7475
	Butyric Acid	27.35 ± 11.81	32.13 ± 18.52	4.775 ± 16.91	0.0449	31.14 ± 14.91	30.17 ± 24.54	−0.976 ± 22.51	0.6940	0.2047
		**LL Group (*n* = 18)**				**Placebo Group (*n* = 17)**				
Indole-derived metabolites (peak area)	5-HIAA	2.14 × 10^−2^ ± 1.77 × 10^−2^	3.18 × 10^−2^ ± 3.33 × 10^−2^	1.04 × 10^−2^ ± 3.45 × 10^−2^	0.2462	4.25 × 10^−2^ ± 4.09 × 10^−2^	2.37 × 10^−2^ ± 3.85 × 10^−2^	−1.88 × 10^−2^ ± 4.98 × 10^−2^	0.0448	0.0224
	Indole-3-lactic Acid	1.83 × 10^−3^ ± 3.29 × 10^−3^	2.76 × 10^−3^ ± 3.84 × 10^−3^	0.93 × 10^−3^ ± 5.44 × 10^−3^	0.2842	5.72 × 10^−3^ ± 18.8 × 10^−3^	0.89 × 10^−3^ ± 1.08 × 10^−3^	−4.83 × 10^−3^ ± 1.81 × 10^−2^	0.0150	0.0209
	Indole-3-glycol	1.40 × 10^−3^ ± 2.45 × 10^−3^	2.21 × 10^−3^ ± 3.09 × 10^−3^	0.81 × 10^−3^ ± 2.58 × 10^−3^	0.0505	1.00 × 10^−3^ ± 1.64 × 10^−3^	1.09 × 10^−3^ ± 2.32 × 10^−3^	0.09 × 10^−3^ ± 2.80 × 10^−3^	0.5791	0.0487
	Tryptophan	3.29 × 10^−1^ ± 2.60 × 10^−1^	2.66 × 10^−1^ ± 1.82 × 10^−1^	−0.63 × 10^−1^ ± 3.17 × 10^−1^	0.5798	2.93 × 10^−1^ ± 3.30 × 10^−1^	2.30 × 10^−1^ ± 1.24 × 10^−1^	−0.63 × 10^−1^ ± 3.41 × 10^−1^	0.9265	0.5905
	Indole Aldehyde	1.14 × 10^−2^ ± 1.17 × 10^−2^	9.07 × 10^−3^ ± 6.82 × 10^−3^	−2.31 × 10^−3^ ± 1.07 × 10^−2^	0.6095	8.99 × 10^−3^ ± 7.68 × 10^−3^	5.84 × 10^−3^ ± 3.66 × 10^−3^	−3.15 × 10^−3^ ± 7.94 × 10^−3^	0.2842	0.6598
	Indole-3-acetic Acid	3.65 × 10^−3^ ± 6.25 × 10^−3^	3.15 × 10^−3^ ± 3.52 × 10^−3^	−0.50 × 10^−3^ ± 7.58 × 10^−3^	0.5019	2.06 × 10^−3^ ± 1.89 × 10^−3^	3.12 × 10^−3^ ± 3.87 × 10^−3^	1.05 × 10^−3^ ± 4.40 × 10^−3^	0.4238	0.8768
	Indole-3-propionic Acid	3.20 × 10^−2^ ± 2.47 × 10^−2^	2.35 × 10^−2^ ± 1.91 × 10^−2^	−8.50 × 10^−4^ ± 2.94 × 10^−2^	0.3038	2.26 × 10^−2^ ± 2.15 × 10^−2^	2.67 × 10^−2^ ± 2.31 × 10^−2^	4.10 × 10^−4^ ± 3.31 × 10^−2^	0.5791	0.2447

Values are presented as mean ± SD. ^1^ Analyzed by Wilcoxon signed rank test between baseline and 12 weeks within each group. ^2^ Analyzed by Wilcoxon rank sum test between the groups at change value.

## Data Availability

The datasets generated and analyzed during the current study are available from the corresponding author on reasonable request. The data are not publicly available due to privacy and ethical restrictions.

## Supplementary Materials

The following supporting information can be downloaded at https://www.mdpi.com/article/10.3390/nu17203313/s1: Table S1: Changes in laboratory tests before and after 12 weeks of intake.
