# Supplementary material for: Efficacy and Safety of Lactobacillus delbrueckii subsp. lactis CKDB001 Supplementation on Cognitive Function in Mild Cognitive Impairment: A Randomized, Double-Blind, Placebo-Controlled Clinical Trial
Source: Nutrients. 2025 Oct 21;17(20):3313. doi: 10.3390/nu17203313 (PMC12567422; doi:10.3390/nu17203313)
Supplement: Supplementary file 1 [file nutrients-17-03313-s001.zip › nutrients-3910192-supplementary.pdf]

Table S1. Changes in laboratory tests before and after 12 weeks of intake.

		LL group (n=50)				Placebo group (n=50)				*p*-value[3)]
		Baseline	12 week	Change Value	*p*-value[1)]	Baseline	12 week	Change Value	*p*-value[1)]	
CBC	Hemoglobin (g/dL)	13.35±1.04	13.15±1.10	-0.19±0.51	0.015	13.59±1.11	13.39±1.14	-0.21±0.55	0.0018[2)]	0.6438[4)]
	Hematocrit (%)	40.34±3.30	39.56±3.59	-0.73±2.04	0.0165	41.10±3.54	40.52±3.35	-0.66±1.70	0.0115[2)]	0.9795[4)]
	WBC (10^3/μL)	5.68±1.75	5.62±1.69	-0.07±1.34	0.4295[2)]	5.59±1.64	5.52±1.81	-0.12±1.35	0.2027[2)]	0.7666[4)]
	RBC (10^6/μL)	4.25±0.38	4.19±0.41	-0.06±0.20	0.0556	4.31±0.41	4.27±0.40	-0.04±0.19	0.0537[2)]	0.7415[4)]
	Platelet (10^3/μL)	225.70±48.19	223.35±50.00	-1.54±23.68	0.654	211.50±51.85	212.88±58.52	1.50±35.21	0.7692	0.6208
	Neutrophils (%)	49.29±7.98	50.47±8.77	0.76±7.86	0.6492[2)]	49.19±8.39	48.62±9.21	-0.31±6.25	0.9960[2)]	0.6896[4)]
	Lymphocyte (%)	39.67±7.11	38.66±7.96	-0.73±6.91	0.5042[2)]	39.69±7.57	40.41±8.51	0.46±5.90	0.8597[2)]	0.6815[4)]
	Monocyte (%)	7.63±2.57	7.47±2.46	-0.02±1.11	0.8763	7.93±1.75	7.81±1.72	-0.06±1.20	0.7197	0.8738
	Eosinophils (%)	2.59±2.02	2.52±1.98	-0.06±1.47	0.7307[2)]	2.37±1.78	2.34±1.57	-0.09±1.09	0.5808	0.7470[4)]
	Basophils (%)	0.83±0.35	0.88±0.39	0.05±0.29	0.0718[2)]	0.82±0.39	0.82±0.43	-0.01±0.26	0.6942[2)]	0.1178[4)]
Biochemistry	AST (U/L)	26.18±6.79	25.23±5.81	-0.77±3.64	0.1489	27.60±6.50	27.48±6.53	0.46±5.39	0.5584	0.1939
	ALT (U/L)	23.08±9.17	21.00±6.92	-1.65±7.73	0.0912[2)]	23.54±10.66	22.83±8.55	0.42±8.32	0.8111[2)]	0.2148[4)]
	ALP (U/L)	133.88±75.92	134.46±78.67	-0.17±17.81	0.7347[2)]	127.10±68.84	129.21±65.66	-0.81±26.55	0.9668[2)]	0.8719[4)]
	Total cholesterol (mg/dL)	195.16±40.39	187.06±40.73	-7.46±32.35	0.1108[2)]	198.24±44.03	189.21±39.79	-8.71±23.52	0.0136	0.5142[4)]
	LDL-C (mg/dL)	116.68±40.64	111.22±35.45	-5.16±30.45	0.3656[2)]	119.76±37.92	111.81±33.55	-7.98±21.62	0.0138	0.3872[4)]
	HDL-C (mg/dL)	58.00±10.97	57.04±11.83	-0.50±7.43	0.6432	57.00±14.08	55.17±14.08	-2.06±8.65	0.0382[2)]	0.3183[4)]
	Triglyceride (mg/dL)	126.54±67.23	108.85±54.05	-19.79±63.21	0.0073[2)]	128.58±70.53	123.88±67.89	-5.44±54.48	0.4583[2)]	0.2669[4)]
	Albumin (g/dL)	4.39±0.22	4.38±0.22	-0.00±0.24	0.6483[2)]	4.43±0.23	4.38±0.25	-0.05±0.20	0.0682	0.2924[4)]
	Total protein (g/dL)	7.31±0.42	7.25±0.35	-0.06±0.28	0.126	7.36±0.36	7.30±0.29	-0.06±0.31	0.0772[2)]	0.8166[4)]
	Total bilirubin (mg/dL)	0.79±0.21	0.81±0.22	0.02±0.15	0.2629	0.85±0.26	0.85±0.34	0.01±0.22	0.9294[2)]	0.3908[4)]
	Glucose (mg/dL)	101.28±9.80	102.17±10.06	0.29±8.19	0.8063	98.94±11.19	99.27±9.99	0.40±7.87	0.7292	0.9495
	BUN (mg/dL)	16.00±3.08	15.94±3.79	-0.13±3.69	0.8062	15.89±3.73	15.07±4.16	-0.74±3.76	0.1772	0.4224

	Creatinine (mg/dL)	0.82±0.17	0.84±0.17	0.01±0.07	0.1579	0.83±0.17	0.84±0.16	0.02±0.11	0.2128	0.9853[4)]
	Uric Acid (mg/dL)	4.63±1.21	4.76±1.27	0.12±0.53	0.1156	4.71±1.32	4.83±1.31	0.15±0.74	0.1767	0.8616
	P (mg/dL)	3.68±0.45	3.65±0.41	-0.01±0.35	0.5081[2)]	3.66±0.47	3.72±0.50	0.04±0.52	0.5662	0.5593[4)]
	Ca (mg/dL)	9.30±0.42	9.38±0.41	0.10±0.44	0.1288	9.31±0.64	9.37±0.54	0.09±0.61	0.3422[2)]	0.5895[4)]
	Na (mmol/L)	141.55±2.92	141.21±2.40	-0.30±2.53	0.413	141.05±2.73	141.17±2.66	0.08±2.61	0.8388	0.4721
	K (mmol/L)	4.18±0.29	4.16±0.28	-0.01±0.32	0.8159	4.27±0.27	4.20±0.36	-0.07±0.39	0.0834[2)]	0.3031[4)]
	Cl (mmol/L)	103.55±2.21	103.92±1.95	0.36±2.65	0.3536	103.10±2.33	103.92±2.33	0.88±3.44	0.0849	0.4122
Urinalysis	pH	6.03±0.87	5.90±0.87	-0.10±0.98	0.4644	6.06±0.99	6.00±1.09	-0.05±1.04	0.6461[2)]	0.9224[4)]
	Specific gravity	1.02±0.01	1.02±0.01	0.00±0.01	0.4653[2)]	1.02±0.01	1.02±0.01	-0.00±0.01	0.5795[2)]	0.2146[4)]

Values are presented as mean ± SD. [1)]Analyzed by paired t-test between baseline and 12 weeks within each group. [2)]Analyzed by Wilcoxon signed rank test between baseline and 12 weeks within each group. [3)]Analyzed by two-sample t-test between the groups at change value. [4)]Analyzed by Wilcoxon rank sum test between the groups at change value.
